# The impact of internet use frequency on non-suicidal self injurious behavior and suicidal ideation among Chinese adolescents: an empirical study based on gender perspective

**DOI:** 10.1186/s12889-020-09866-0

**Published:** 2020-11-16

**Authors:** Xueyan Yang, Moye Xin, Kun Liu, Bilun Naz Böke

**Affiliations:** 1 Institute for Population and Development Studies, Xi’an Jiaotong University, No.28, Xianning West Road, Xi’an, 710049 Shaanxi China; 2 Human Development, McGill University, 3700 McTavish Street, Montreal, QC H3A1Y2 Canada

**Keywords:** NSSI, Suicidal ideation, Internet use frequency, Adolescents, Gender difference

## Abstract

**Background:**

We attempted to find if there were gender differences in Non-suicidal self injurious (NSSI) behaviors and Suicidal ideation among Chinese adolescents, then analyze the impact of Internet use frequency on these variables among adolescents of different genders.

**Methods:**

Based on the data from 6 high-schools and 4 universities in 4 cities in China, the gender difference in NSSI behaviors and Suicidal ideation and their related factors were analyzed in the study.

**Results:**

Gender differences were found during different purposes of Internet use; There was no significant gender difference in NSSI behaviors among Chinese adolescents, yet females reported significantly higher intensity of suicidal ideation compared to males; Internet use frequency could explain the prevalence of NSSI behaviors and Suicidal ideation by gender, to some categories.

**Conclusions:**

There were gender differences in Internet use frequency among adolescents; Gender difference of NSSI engagement among Chinese adolescents was not statistically significant; Females had higher suicidal ideation than males; the overuse of social softwares was found to be a risk factor to both NSSI engagements and suicidal ideations for both genders; males would engage less NSSI behaviors when they spent more time on knowledge sharing softwares while might have more suicidal ideation when they spent too much time on gaming.

## Background

Non-suicidal self-injury (NSSI) and suicidal ideation are major health concerns among adolescents worldwide [1–4]. Although suicidal ideation broadly refers to thoughts about dying or wanting to die as well as the formation of plans to die [5], NSSI is distinctive in that the intention is not to die. Specifically, NSSI refers to deliberate self-inflicted damage to one’ s own body tissue by methods such as cutting, scratching, and self-hitting that leads to tissue damage without conscious suicidal intent and for reasons not socially sanctioned [6].

In China, the problem of suicide and NSSI, especially among adolescents, is more severe compared to Western studies [3, 4], yet much of the literature on life threatening behavior stems from research within Western populations [2, 7]. This has resulted in much of the present theory on life threatening behaviors as well as the clinical implications to be based on Western populations, which largely ignores the nuances found in other cultural contexts [8].

A study surveying nearly 1000 university students in China revealed that 35.2% have engaged in NSSI behaviors in their lifetime [9], which is nearly double the prevalence rate among university students from the U.S. and Canada [10]. Furthermore, the prevalence of NSSI in China appeared quite higher among adolescents, where research based on a research sample of 2120 middle school students has found that 57.4% have engaged in NSSI behaviors [11]. This study also suggests a much higher rate of engagement in NSSI over the past year and an earlier age of onset relative to much of the research from Western contexts [9–11].

Meanwhile, relevant studies have also revealed the seriousness of suicidality in Chinese adolescents can not be ignored. The results of suicidal ideation and attempted suicide behavior surveys published after the year 2010 in different cities of China showed that the annual prevalence of suicidal ideation was between 13.2–28.0%, and the prevalence of attempted suicide was between 1.2–4.0% [12–14]. Moreover, Gao conducted a survey among 2416 primary and middle school students in Shanghai and found that the prevalence of suicidal ideation was 15.23%, suicide plan was 5.84%, and attempted suicide was 1.74% [15]. Unlike most countries where the suicide rate of male adolescents is higher than that of females, the characteristics of suicidal behavior among Chinese adolescents were found to be somewhat different whereby the suicide rate is higher among females compared to males, and female adolescents report more suicidal ideation than male adolescents [12–15].

As such, there is a need for further investigations aimed at better understanding NSSI and suicidality among Chinese adolescents, and the possible mechanisms that might contribute to both increased suicidality and greater endorsement of NSSI behaviors. Several studies have found excessive Internet use to be associated with both NSSI and suicidality among young people [16–19], however, the exact mechanism by which Internet use contributes to either NSSI or suicidality remains unclear.

### A link between internet use frequency and NSSI/suicidal ideation among adolescents

Internet use is especially frequent and widespread among adolescents both in China and the United States, with about 80% of adolescents using the Internet regularly [19, 20]. Adolescents are increasingly relying on the Internet as a primary mode of communication, whether for emails or for social softwares. One particular purpose might be for support from others through discussion forums or private messaging. According to the results of a study based on 856 adolescents’ Internet use frequency and preference, almost all adolescents have used the Internet to fufill their social and daily needs. Only 4 of them have never used any online media, and 852 people have actually used online media as a daily communication tool, accounting for 99.53%. Among the many ways of using Internet, social software is the most popular among adolescents, and more than half of them have used social software (474, 55.63%). Among them, 340 male users accounted for 73.27%, followed by Instant Messaging (IM) software (392 people, 46.01%), Video software (374 people, 43.90%), Shopping software (392 people, 46.01%))Knowledge Sharing software (374 people, 43.90%), however, relevant research did not report whether gender differences exist in the above data [21]. Furthermore, mutiple studies also indicated that no significant difference was found in Internet usage preference among different age groups [21–24].

Theoretically speaking, this developmental phenomenon might be explained in two opposite sides: the potential benefits of the Internet the Internet itself provides a powerful resource for young people who want information on socially sensitive topics, such as sexual perplexity, interpersonal relationships and NSSI/Suicidal related issues [25]. This form of communication style is specifically beneficial for shy, socially anxious or marginalized young people, enabling them to improve their social communication skills without having to take the risks associated with “face to face” interactions [26, 27]. In addition, online communication may encourage more authentic communication, many adolescents admit that they are more willing to share their “real thoughts and feelings” online than when they are in real-life communication [28]. Obviously, the Internet is impacting on the social environment of adolescents, by affecting their ways of communication, establishment and maintenance of interpersonal relationship, and seeking for social support.

Notwithstanding the potential benefits of the Internet such as making communication more accessible, research has found the excessive reliance upon the Internet to be associated with psychological and physical harms (e.g., isolated, extreme and poor communication). For instance, several studies among Chinese middle school students who engaged in excessive Internet use reported adverse physical consequences such as shoulder pain caused by prolonged poor posture when sitting, decreased visual perception loss of appetite, decreased sleep quality as well as decreases in immune function [28]. Students also reported psychological consequences including difficulties with self-control, more social avoidance and negative coping [29], increased levels of perceived emotional and social loneliness which lead to lose oneself in social role [30].

Furthermore, research has focused on the effect of Internet use may have on the likelihood of adolescents engaging in life threatening behaviors [31]. Research suggests that adolescents who experience suicidal thoughts or engage in NSSI behaviors appear to use the Internet to seek out specific discussion forums for support [32]. A review of the Chinese instant messaging software QQ revealed that the popular online platform was hosting more than 600 groups pertaining to suicide and self-injury [33]. However, these forums and online spaces are often not monitored or moderated therefore individuals can be exposed to inaccurate and/or harmful information about suicide and NSSI [34]. As a result, the openness, virtuality and exemption of the Internet make information about NSSI or suicide online easily accessible [35], and discussing online with people who are also interested in suicide and NSSI related topics may lead to the encouragement of suicide and NSSI [16, 36]. According to Messias and her colleagues, daily use of the Internet for more than 5 h was closely related with higher levels of depression and suicidality (both ideation and attempts) among adolescents [34]. Another critical phenomenon is that adolescents with potential NSSI thoughts or suicidal ideation might search the Internet for contents about NSSI or suicide related information. For example, those people who have the history of engaging in NSSI or suicidal behavior can easily connect with people who are now engaging in these health risk behaviors, while those who are curious about NSSI and suicidal behaviors will directly get linked to a unprotected world of content about NSSI and suicidal behaviors [16]. Thus, these related studies above indicate that excessive Internet use could be a risk factor for adolescents’ engagement in NSSI and suicidal behaviors [16, 36–38]. Therefore, the Internet may play as a powerful double-edged sword in supporting individuals’ suicidality as well as their endorsement of NSSI behaviors.

Besides, although NSSI behaviors and suicidal ideation are two completely isolated and different concepts, their differences varied in intention, lethalities, methods, cognition, and results [39], studies have revealed that there is a highly similarity and inherent compatibility between NSSI behaviors and suicidal ideation. Firstly, individuals who had history of NSSI or suicidal ideaion had the same psychological characteristics, which may be a negative response to bad emotions, an extreme feedback to various problems in the growing stage, or a means to attract attention and obtain a sense of existence. Secondly, NSSI behaviors can be an effective power of avoidance and control against suicidal ideation [40]. Thirdly, a large number of empirical data showed that there are links existing between NSSI and suicidal ideation. Laye-Gindhu conducted a survey among 424 adolescents in Canada, found that the intensity of suicide ideation among NSSI group was significantly higher than that of non NSSI group [41]. Meanwhile, 89% of the respondents who attempted suicide had experience of NSSI [42]; other research pointed out that many people who had NSSI also had suicidal ideation in the past [43, 44]. Finally, Firestone analyzed a negative thought pattern aiming at terminating the coherence of suicidality, then confirmed that there was a direct relationship between NSSI, suicidal ideation and the negative thought pattern [45]. Moreover, studies have found that different demographic variables (e.g., age, only-child, parents’s educational level, etc) had striking links between individual’s Internet usage and life-threatning behavior (e.g., NSSI, Suicidal ideation, etc). According to related studies, elder adolescents in Senior high school with higher reliance on the Internet seemed to have a higher possiblity of transformation of Internet addiction, which could be a risk factor to individual’s life-threatning behavior, comparing to those younger adolescents in Junior high school [46]; In Wang’s study, she claimed that with less family emotional care and attention, parents’ divorce and lower education level of parents would increase the frequency on adolescents’ Internet usage, which could directly increase the risk level of their Internet addiction, causing higher possibility of life-threatning behavior to emerge [28]; Additionally, several Chinese studies have found that, compared with the only child, the non only child will receive less emotional care and attention from their parents, which will lead to more free time for them to have on-line activities, thus creating a higher possibility of Internet addiction, which also could be threatening to their physical and mental health [22, 47]. Thus, it is necessary to analyze NSSI behaviors and suicidal ideation as common dependent variables to study the impacting mechanism of Internet use frequency and other demograhic variables with them furthermore.

### The gaps

Relevant studies claimed that no significant gender difference was found during the influential process of traditional media (e.g., Radio, TV programs, music, magazines, movies, etc) on adolescents’ life-threatening behaviors [48], and massive evidence has proved that media violence accompanied by traditional media could be a risk factor contributing to the increasing numbers of agressive behaviors (e.g., NSSI, etc) among adolescents [49]. The Internet is a relatively dynamic and burgeoning media but has the same concern. Although Western literature hints that Internet use may be important variable to study when examining NSSI and suicidal behaviors [50], little is known about the role of Internet use frequency in NSSI and suicidal behaviors among Chinese adolescents. The consequences of Internet use frequency as a risk factor for NSSI engagement and suicidal thoughts and/or behaviors among Chinese adolescents remains to be investigated.

Meanwhile, findings using gender analysis reported gender differences in the prevalence of NSSI. The prevalence of NSSI behavior among female adolescents was higher than that of males, and therefore was viewed as a “feminine” behavior [51–53]. A recent meta-analysis also found a female bias in NSSI prevalence among adolescents worldwide [54].

For suicidality, Kõlves’s team found that in comparison with female adolescents, the risk of suicidal ideation among males has increased in recent years [55]. According to Freeman and colleagues, suicidal ideations were rated significantly more frequently in males than females [56].

Recent research on these topics in China adolescents haven’t paid much attention on gender differences in the context of Internet use frequency, NSSI and suicidal ideation. Since NSSI and suicidality have always been the forefront of related psychological, sociological and demographic research [57–62], which helps to explain this gender bias, it is suggested that special attention should be paid to whether there are gender differences in the relation between Internet use frequency, NSSI and suicidal ideation under Chinese context.

Thus, the overarching objective of this paper is to examine the relation between gender, Internet use frequency, NSSI, and suicidal ideation among adolescents in China. Specifically, this study seeks to (1) assess whether any gender differences in Internet use frequency, NSSI and suicidal ideations exist among adolescents; (2) whether Internet use frequency will act as a risk factor for NSSI and suicidal ideation engagement within this group.

Three hypotheses associated which each of the 2 objectives above are as follows:

- H1: There will be gender differences in different kinds of Internet use frequency.
- H2: NSSI engagement and suicidal ideation will be more prevalent among females.
- H3:Different categories of Internet use frequency will all positively predict engagement in NSSI and suicidal ideation among adolescents indicating them as risk factors.

## Methods

### Participants

Participants consisted of a total of 2018 middle-school and university students (803 males, 1215 females, *M*_*age*_ = 17.8 years, age range: 12–24 years) who were recruited to complete questionnaires from 6 middle-schools and 4 universities in Xi’an, Yulin, Ankang of Shaanxi Province and Binzhou of Shandong Province in China. Parents or legal guardians gave permission for students’ participation by signing consent forms for those who were younger than 18. The study was approved by the institutional ethics review board within the university and the schools where the survey was conducted.

### Measures

#### NSSI status

In order to assess adolescents‘engagement in NSSI behavior, the Non-suicidal Self-Injury Assessment Tool (NSSI-AT) [63], was selected and translated into Chinese. By asking, “have you ever done anything that you didn’t intentionally harm yourself for the purpose of suicide?”, measured as “0 = no, 1 = yes”. Based on the 14 kinds of NSSI behaviors stipulated by NSSI-AT, this study refers to the relevant research of adolescents in the Chinese social and cultural environment, adolescents tend to engage in different methods of self-injury from Western samples [64], including “1. Swallowing items that cannot be digested, such as plastic, stone, etc; 2. Taking or swallowing too much medicine (beyond the medical advice); 3. Burn yourself with cigarette butts “, etc. After adding 4 kinds of specific NSSI behaviors, a total of 18 specific NSSI behaviors was defined. Coefficients obtained by looking at agreement on a number of NSSI-AT variables and scales indicated that NSSI-AT scores exhibited preliminary reliability in campus population. Moreover, the relevancy of internal consistency is quite significant and has been proved to be practical and effective by relevant research subsequently. The internal consistency of this scale was found to be adequate for the present study (α = 0.82).

#### Suicidal ideation

The Scale of Suicidal Ideation (SSI) developed by Beck [65] was used to assess adolescents’ suicidal behaviors in the last 12 months. This measure consists of 14 items measuring participants’ suicidal ideation (e.g., I think suicide can end the current pain, I imagined about taking some strange or dangerous drugs to suicide on purpose, etc) on a 5-point Likert scale from disagree to completely agree. Higher scores indicate higher intensity of suicidal ideation. The SSI (α = 0.81) showed great reliability.

#### Internet use frequency

Emotional Health Online Behavior Assessment (EHOBA), developed by De Riggi, Lewis and Heath [66], was used to assess Internet use frequency among adolescents. This scale consists of 6-items measuring one’s use of the Internet for different categories, items were modified to better fit the Chinese context. Items include: “How often do you use IM softwares (e.g., Wechat, QQ, Messenger, etc)? ”, “How often do you use social softwares (e.g., Weibo, Twitter, etc)? ”, “How often do you use a video site? (e.g., Youku, Youtube, Bilibili, etc)? ”, “How often do you use the shopping website? (e.g., Taobao, Amazon, etc)? ”, “How often do you use knowledge sharing software? (e.g., Zhihu, Reddit, Google scholar, etc)? ”, “How often do you play online games (Fortnite, Call of Duty, etc)? ”, Participants were asked to rate each item on a 5-point Likert scale from “never use” to “use it everyday” whereby higher scores indicating higher Internet use frequency. The EHOBA was found to have excellent internal reliability in the present study (α = 0.91).

### Procedure

The present study employed a combination of convenience sampling to recruit teachers within 6 high-schools and professors within 4 universities who expressed interest in having their students participate in this study. Within the schools, stratified sampling was used to recruit participants to complete measures to ensure equal representation of gender as well as different grade levels within middle school (from grade 2 in middle-school to grade 4 in university). All questionnaires were anonymous. A total of 2400 questionnaires were distributed and 2018 valid questionnaires were recovered therefore the consent rate in the present study was 87.83%.

### Data analysis

Chi-square test and independent sample t-tests were adopted to analyze the gender differences in Internet use frequency, NSSI prevalence and suicidal ideation intensity among adolescents.

Furthermore, a step-wise binary logistic regression was used to examine whether Internet use frequency (Step 1), and demographic variables (Step 2) were predictive of NSSI behavior (Model 1–4) for both genders, also a step-wise linear regression was used to examine whether the independent variables above were predictive of suicidal ideation (Model 5–8) for both genders.

## Results

The results of Gender differences of Internet use frequency among adolescents is presented in Table 1. Firstly, gender differences were found during different purposes of Internet use. Male students tend to spend more time using knowledge sharing softwares and gaming softwares than females (*M* = 4.12, 3.33, *p <* 0.01), yet female students tend to spend more time using IM, social and shopping softwares than male students (*M* = 4.49, 4.42, 3.67, *p <* 0.001); Secondly, A total of 374 participants reported engaging in NSSI indicating a prevalence rate of 18.5%. Prevalence rates were found to be 17.2 and 19.4% among males and females, respectively. Chi-square test results showed that there was no significant difference in the prevalence of NSSI between male and females (χ^2^ = 1.226, *df* = 6, *p* > 0.1). Additionally, we found that the suicidal ideation intensity of females (*M* = 101.05) was significantly (*χ2* = 3.104, *p <* 0.001) higher than that of males (*M* = 88.37).

**Table 1:** Gender differences of Internet use frequency among adolescents

Internet Use Frequency	Male(803)		Female(1215)	
	M	*SD*	M	*SD*
IM softwares	4.39	3.64	4.49	3.13
*t*	*t* = 5.78***			
Social softwares	4.13	2.74	4.42	2.80
*t*	*t* = 8.93***			
Video softwares	4.07	3.12	4.14	3.28
*t*	*t* = 8.35			
Shopping softwares	3.04	0.74	3.67	0.90
*t*	*t* = 9.34***			
Knowledge sharing softwares	4.12	1.87	2.61	0.86
*t*	*t* = 3.03**			
Online gaming	3.33	0.85	2.09	1.07
*t*	*t* = 4.70**			

Note. + , p < 0.1;*, p < 0.05;**, p < 0.01;***, p < 0.001

Table 2 presents the results from binary logistic regression on factors associated with the prevalence of NSSI behaviors among adolescents by gender. Model 1 revealed that the use of social softwares positively predicted NSSI engagement among males (social softwares: EXP(β) = 0.786, *p* < 0.05), while the use of knowledge sharing softwares negatively predicted NSSI engagement among males (knowledge sharing softwares: EXP(β) = − 1.091, *p* < 0.01); When control variables were introduced to model 2, the use of social and knowledge sharing softwares became even more significant, meanwhile, only-child from control variables had significant negative impact on NSSI behaviors among males (only-child: EXP(β) = − 0.746, *p* < 0.01).

**Table 2:** The Impact of Internet Use Frequency on the Prevalence of NSSI Behavior Among adolescents by Gender

Dependent: whether NSSI behavior occurs (reference: No)		Males		Females	
		Model 1	Model 2	Model 3	Model 4
Independent Variable: Internet use frequency	IM softwares	0.054	0.985	0.912	1.024
	Social softwares	0.786*	1.012**	0.993***	1.025***
	Video softwares	1.034	1.055	0.972	0.860
	Shopping softwares	0.985	−0.900	1.139	1.016
	Knowledge sharing softwares	−1.091**	− 1.097***	0.994	0.984
	Online gaming	0.927	1.840	1.307	0.950
Control variables	Age		1.030		−0.886
	Only-child (reference: No)		−0.746**		−1.117*
	Father’s educational level (reference:primary school and below) middle school or above		−0.793		−1.125
	Mother’s educational level (reference:primary school and below) middle school or above		−1.279		−1.089
	Parents’ marital status (reference:separated) Married		−0.762***		−0.969**
95% CI		0.73, 0.86**	0.84, 1.05**	1.22, 0.94**	1.30,1.10**
−2 Log Likelihood		607.07**	591.36***	787.32***	845.21***
Cox & Snell R^2^		0.003	0.023	0.002	0.025
Nagelkerke R^2^		0.005	0.037	0.004	0.040

Note. + p < 0.1;*p < 0.05;**p < 0.01;***p < 0.001

As the variables were included into the models step by step, their explanatory power increasingly improved. The Cox & Snell R^2^ and Nagelkerke R^2^ in Model 1 were only 0.003 and 0.005, respectively, while in Model 2, this markedly improved to 0.023 and 0.037 when control variables were included.

In model 3, the use of social softwares positively predicted NSSI engagement among females (social softwares: EXP(β) = 0.993, *p* < 0.001). After control variables were included in model 4, the use of social softwares remains significant, only-child variable negatively predicted NSSI behaviors among females (only child: EXP(β) = − 1.117, *p* < 0.05).

As the variables were included into the models step by step, their explanatory power increasingly improved. The Cox & Snell R^2^ and Nagelkerke R^2^ in Model 3 were only 0.002 and 0.004, respectively, which improved to 0.025 and 0.040 when control variables were added in model 4.

Table 3 presents the results from linear regression on factors associated with suicidal ideation intensity among adolescents by gender. In model 5, the use of social softwares and online gaming positively predicted suicidal ideation intensity among males (social softwares: EXP(β) = 0.037, *p* < 0.001; online gaming: EXP(β) = 0.124, *p* < 0.05); When control variables were introduced to model 6, the significance of social softwares and online gaming remained almost unchanged in the coefficient size and significance as compared to Model 5; meanwhile, age from control variables negatively predicted suicidal ideation intensity among males (age: EXP(β) = − 0.212, *p* < 0.001).

**Table 3:** The Impact of Internet Use Frequency on the Intensity of Suicidal Ideation Among adolescents by Gender

Dependent: Suicidal Ideation Intensity		Males		Females	
		Model 5	Model 6	Model 7	Model 8
Independent Variable: Internet use frequency	IM softwares	−0.175	−0.188	0.012	0.052
	Social softwares	0.037***	0.046***	0.163*	0.159**
	Video softwares	−0.008	−0.041	0.038	0.033
	Shopping softwares	0.077	0.004	0.128	0.082
	Knowledge sharing softwares	−0.031	−0.045	−0.036	− 0.021
	Online gaming	0.124*	0.119*	0.071	0.087
Control variables	Age		−0.212***		−0.135*
	Only-child (reference: No)		0.037		0.003
	Father’s educational level (reference:primary school and below) middle school or above		0.073		−0.016
	Mother’s educational level (reference:primary school and below) middle school or above		0.109		−0.046
	Parents’ marital status (reference:separated) Married		−0.163***		−0.046**
95% CI		0.61, 0.91***	0.75, 1.03***	1.03, 0.74**	1.26,0.93**
F		3.255***	5.133***	2.132**	2.655***
df		723	723	1187	1187
Adjusted R^2^		0.14	0.45	0.08	0.33

Note. + p < 0.1;*p < 0.05;**p < 0.01;***p < 0.001

As the variables were included into the models step by step, their explanatory power increasingly improved. The adjusted R^2^ in Model 5 was only 0.14, respectively, while in Model 6, this markedly improved to 0.45 when control variables were included.

In model 7, the use of social softwares positively predicted suicidal ideation intensity among females (social softwares: EXP(β) = 0.163, *p* < 0.01). After control variables were included in model 8, the significance of the use of social softwares remained almost unchanged in significance as compared to Model 7 (social softwares: EXP(β) = 0.159, *p* < 0.01). Meanwhile, age from control variables negatively predicted suicidal ideation intensity among females (age: EXP(β) = − 0.135, *p* < 0.05).

As the variables were included into the models step by step, their explanatory power increasingly improved. The adjusted R^2^ in Model 7 was only 0.08, respectively, while in Model 8, this markedly improved to 0.33 when control variables were added.

## Discussion

Our analysis indicated that gender differences were found during different purposes of Internet use. Male students tend to spend more time using knowledge sharing softwares and gaming softwares than female students. This finding is consistent with existing research, on the basis of a meta-analysis of the user composition of Zhihu, a popular online knowledge sharing platform in China, among the 30,000 users, 72.4% are male users and 27.6% are female users [67], which clearly demostrated the proportional advantage of male adolescents in this area. Meanwhile, relevant study showed that male adolescents used online games more frequently than women, which coincides with the results of this study. One possible explaination could be that, comparing to female adolescents, male adolescents were more dependent on online games to relieve the pressure of daily life, plus there were few cases of female online game addicts reported [48]. On the other hand, female students tend to spend more time using IM, social and shopping softwares than male students. On this finding, we haven’t found relevant empirical research for support. But this could probably be explained by the theory of “Gender pattern” aroused by Zhou, which indicates that females are more willing to engage in frequent social interaction and communication based on different platforms than males, at the mean time, the enjoyable shopping experience makes it easier for women to get great spiritual satisfaction and not hesitate to spend more money and time to enjoy the pleasure than males [68].

The results revealed that the prevalence of NSSI behaviors was similar among both males (17.2%) and females (19.4%), yet no significant gender difference was found. This finding is consistent with studies in other contexts [9, 69]. Existing studies in western countries stated that the gender difference in the prevalence of NSSI among clinical samples is more signicant than that in community samples [54], but our analysis results are different: there is no gender difference in the prevalence of NSSI among clinical samples. However, given the limited number of studies were included in clinical samples (only six), this result should be interpreted with extra caution.

Meanwhile, we found that females reported higher intensity of suicidal ideation comparing to males’ reports. This finding is in accordance with existing studies [70, 71] indicating that females have a greater risk of suicidal ideation. This finding also suggests that China is the only country in which the suicide rate among females is higher than males [72, 73]. This finding is also aligned with studies that have strong links between depressive symptomatology and suicidal ideation [74, 75]. One possible explanation is that Chinese Confucian culture has different expectations for gender roles of men and women. Nowadays in China, families generally believe that girls should be born rich and should be given more care, but boys should be given low support and more strict discipline for a better future. Based on this belief, boys usually face more strict requirements and rules, thus their psychological endurance and tolerance are significantly stronger than girls of the same age, which would results in girls facing life pressure and difficulties, poor tolerance might leads to frequent suicidal ideation [75]. However, this inference should be verified with more data in the future.

The prevalence of NSSI engagement among adolescents was significantly associated with the frequency of certain types of Internet use. Specifically, the use of social softwares had significantly predicted higher prevalence of NSSI engagement among both male and females, that is, individuals who spend more time on social softwares were more likely to engage NSSI behaviors. This shows that over dependence on social softwares can be a risk factor for adolescents’ engagement in NSSI. This finding might be explained by the stigma associated with NSSI; individuals who self-injure often do not discuss this behavior with others, including their family or friends [16]. Therefore, social softwares can play an important role in meeting individuals’ need for social support and connection [76, 77], including mitigating feelings of social isolation and even encourage healthier behaviours [78]. According to a related study, photos containing NSSI imagery and content pertaining to NSSI are often posted on social softwares, which may have a negative impact on vulnerable audiences [79] such as adolescents. It has been found that it is often the most graphic images of NSSI with higher severity of wounds get the attract greater attention and gain more comments [80]. Harmful effects of these images can include encouragement of NSSI engagement and the popularization of the behavior [81]. This issue can be exacerbated by the challenges experienced by social softwares platforms as they attempt to find novel and more effective methods to moderate this online content [81].

Contrastingly, the use of knowledge sharing softwares significantly predicted lower prevalence of NSSI engagement among males, indicating that males were less likely to engage in NSSI behaviors when they spent more time on knowledge sharing softwares. As mentioned above, a popular online knowledge sharing platform in China called Zhihu, male users accounted for 72.4% and females accounted for 27.6% of users among a sample of 30,000 [67]. This indicates that males are more likely to use this platform compared to females. Meanwhile, according to related studies, young generation often use knowledge sharing softwares such as Reddit, Zhihu, etc., as a way of finding support and validation with regards to their emotional needs and NSSI in particular, which can help individuals connect to others, obtain support, and gain knowledge leading them to find healthier ways to cope [81–83]. Besides, other factors that may relate NSSI and gender, such as adherence to antidepressant medications [84] and psychoactive substance use [85] should also be noted.

The prevalence of suicidal ideation among adolescents was also correlated with some variables of Internet use frequency. Similar to the relationship between the use of social softwares and NSSI behaviors among adolescents, the use of social softwares also positively predicted suicidal ideation among both males and females, which means individuals were more likely to have greater suicidal ideation when they spend more time on social softwares. This demonstrates that excessive use of social softwares may be a risk factor for adolescents’s suicidal ideation as well. Social softwares play as an important platform of interpersonal communication, especially with a high prevalence among young Internet users. In recent years, the negative influence of overusing social softwares on individual psychological and social adaptation has gradually become the focus of researchers, which not only endangers adolescents’s mental health, but also increases the possibility of suicides [86]. One example is the emergence of negative social software that threatens adolescents’s health in the guise of online social networking lately, such as the “Blue whale challenge” taught adolescents with “no value”should commit suicide in the same way as whales kill themselves by stranding [87]. A number of adolescents were exposed to the suicide rules of these life-threatning activities through the negative influence of social softwares [88]. The “Blue whale challenge” might be vanished already, but adolescents still can get access to these similar social softwares to spend their time on which harm adolescents’s mental and physical health in China and other countries [89–92].

Similarly, excessive online gaming was also a positive predictor of suicidal ideation. Although moderate online gaming can be beneficial to brain function, excessive or online gaming addiction is harmful to both mental and physical health among adolescents, which also leads to loneliness which can be associated with suicidal thoughts or even attempts [47].

### Limitations

This study has several limitations. Firstly, this study relied only on self-report questionnaires to examine NSSI behavior and suicidal ideation. Further investigations are needed to understand the contributors to the patterns of adolescents’s engagement in NSSI and suicidal ideation. Secondly, the study is cross-sectional and the relationships between the variables are not causal. Thirdly, effect sizes are quite small so the results should be interpreted with caution. Fouthly, whether other related variables (e.g., depression symptoms or conflicts with parents, etc) could be risk factors to both life-threatning behavior (e.g., NSSI, etc) and Internet usage among adolescents require more empirical analysis; Fifthly, more comprehensive and mature measurement methods are needed to validate the accurateness and applicability of the study.

### Implications and contributions

This is the first evidence-based study revealed differences by gender in the relationship between Internet use frequency, NSSI engagement and suicidal ideation among adolescents in China. The results revealed a gender pattern in the relationship of Internet use frequency with NSSI behaviors and suicidal ideation among Chinese adolescents. We found gender difference in suicidal ideation and different categories of Internet use frequency will change into different protective or risk factors; specifically, females were more likely to have more suicidal ideation, males would engage less NSSI behaviors when they spend more time on knowledge sharing softwares while might have more suicidal ideation when they spend too much time on gaming., The findings will be helpful to enrich existing literature on Internet use frequency, NSSI and suicidal behaviors among Chinese adolescents, and emphasize the need for continued efforts to explore NSSI and Suicidal behaviors across various cultures and societies. Results also emphasized the need for gender-specific interventions for Chinese adolescents.

## Conclusions

To conclude, as hypothesized, gender differences were found during different purposes of Internet use. Male students tend to spend more time using knowledge sharing softwares and gaming softwares than females, yet female students tend to spend more time using IM, social and shopping softwares than male students.

Secondly, gender difference of NSSI engagement among Chinese adolescents is not statistically significant; While females had more suicidal ideation than males as hypothesized.

Thirdly, not as hypothesized, just a few categories of Internet use frequency were sufficient to become risk factors to both NSSI and suicidal ideations engagements among adolescents, specifically, the overuse of social softwares was found to be a risk factor to both NSSI and suicidal ideations engagements for both genders. In addition, males would engage less NSSI behaviors when they spend more time on knowledge sharing softwares while might have more suicidal ideation when they spend too much time on gaming.

## Data Availability

The data that support the findings of this study are available from the Institute for Population and Development Studies at Xi’an Jiaotong University, but ethical restrictions of Xi’an Jiaotong University apply to the availability of these data, which contains privacy variables that might affect the growth of adolescents’ mental health and were used under license for the current study, and so are not publicly available. Data are however available from the corresponding author upon reasonable request and with permission of the Institute for Population and Development Studies at Xi’an Jiaotong University.

## Abbreviations

NSSI: Non-suicidal self-injury
SI: Suicidal Ideation
IUF: Internet Use Frequency
NSSI-AT: Non-suicidal Self-Injury Assessment Tool
SSI: The Scale of Suicidal Ideation
EHOBA: Emotional Health Online Behavior Assessment
